# Spectral-domain optical coherence tomography for evaluating palisades of Vogt in ocular surface disorders with limbal involvement

**DOI:** 10.1038/s41598-021-91999-2

**Published:** 2021-06-14

**Authors:** Ying-Yi Chen, Yi-Chen Sun, Chia-Ying Tsai, Hsiao-Sang Chu, Jo-Hsuan Wu, Huai-Wen Chang, Wei-Li Chen

**Affiliations:** 1 Department of Ophthalmology, National Taiwan University Hospital, No. 7, Chung-Shan South Road, Taipei, Taiwan; 2 Department of Ophthalmology, Taipei Tzu Chi Hospital, The Buddhist Tzu Chi Medical Foundation, New Taipei City, Taiwan; 3 Graduate Institute of Clinical Medicine, College of Medicine, National Taiwan University, Taipei, Taiwan; 4 Department of Ophthalmology, Fu-Jen Catholic University, New Taipei City, Taiwan; 5 School of Medicine, College of Medicine, Fu-Jen Catholic University, New Taipei City, Taiwan; 6 Advanced Ocular Surface and Corneal Nerve Research Center, National Taiwan University Hospital, Taipei, Taiwan; 7 Shiley Eye Institute and Viterbi Family Department of Ophthalmology, University of California, San Diego, La Jolla, CA USA; 8 Department of Ophthalmology, College of Medicine, National Taiwan University, Taipei, Taiwan

**Keywords:** Stem cells, Diseases, Medical research, Stem-cell research

## Abstract

Spectral-domain optical coherence tomography (SD-OCT) has been used to observe the morphology of the palisades of Vogt (POV) with satisfactory resolutions. In this study, we used SD-OCT to examine the microstructure of the POV in ocular surface disorders with limbal involvement. We detect subclinical limbal pathologies based on five parameters, including (1) decreased epithelial thickness, (2) loss of the sharp stromal tip, (3) loss of the smooth epithelial-stromal interface, (4) dilated stromal vessels, and (5) decreased POV density. Eighteen eyes of 10 patients with advancing wavelike epitheliopathy (AWE) and 15 eyes of 9 patients with phlyctenular keratitis/ocular rosacea were recruited. SD-OCT could detect abnormal changes in the POV in 100% of the lesion sites. In presumed-healthy areas of the diseased eyes diagnosed by slit-lamp biomicroscopy, SD-OCT detected abnormal changes in the POV in 100% of the eyes in both groups. In patients with unilateral disease, abnormal changes in the POV were detected by SD-OCT in 50% and 100% of presumed-healthy eyes diagnosed by slit-lamp biomicroscopy in the AWE group and phlyctenular keratitis/ocular rosacea group, respectively. SD-OCT is powerful in detecting POV changes in ocular surface disorders and can provide useful information that cannot be provided by slit-lamp biomicroscopy.

## Introduction

Limbal stem cells are a group of cells located in the basal epithelial layer of the palisades of Vogt (POV) and are involved in maintenance of epithelial homeostasis and corneal clarity^1–3^. The limbus, a transition zone between the peripheral cornea and conjunctiva, has attracted ophthalmologists’ attention because it provides a niche microenvironment for limbal stem cells^4^. Damage to stem cells in the POV may sequentially result in persistent epithelial defects, conjunctival ingrowth, corneal opacity, and severe vision loss^5^. Thus, understanding the detailed microstructure of the POV by using noninvasive modalities is clinically important^6^.

In addition to the widely used slit-lamp biomicroscopy, various noninvasive imaging modalities are used to determine the structure of the POV, including ultrasound biomicroscopy (UBM), in vivo confocal microscopy (IVCM), and anterior segment optical coherence tomography (AS-OCT). The 50-MHz UBM system can penetrate up to 5–6 mm, thus enabling visualization of the structures of the anterior chamber angle, ciliary body, and posterior chamber. However, UBM is limited by being a close-contact immersion technique. Moreover, the 25-μm axial resolution is inadequate to obtain high-quality images of the POV^7,8^. IVCM can provide detailed information regarding the structure of the POV and limbal stem cells. However, a limitation of IVCM is that it can provide only en face images of a limited area. Compared with IVCM, spectral-domain OCT (SD-OCT) with a corneal anterior module long adaptor lens can provide the cross-sectional images of the POV of both radial and tangential orientations with a satisfactory magnification and resolution. Lin et al. used SD-OCT to examine the role of age and regions in the microstructure of POV in healthy eyes^9^. In that study, the “atypical” pattern of the radial cross-sectional POV was detected as the abnormal thickness of the limbal epithelium and the abnormal morphology of the subepithelial stroma underneath the epithelium of the POV. Thus, the detailed microstructure of the POV could be easily determined through SD-OCT^9^. Haagdorens et al. used tangential cross-sectional images obtained by SD-OCT to observe and quantify limbal crypts and the density of POV (presented as number of palisades per millimeter) and provided useful information for evaluation of the POV^10^. Furthermore, studies have used SD-OCT to grade the abnormality of the POV and demonstrated the power of SD-OCT to grade disease severity in limbal stem cell deficiency (LSCD)^11–13^. All these results have demonstrated the value of SD-OCT for evaluation of the POV in diseased eyes.

Although SD-OCT has been increasingly used to observe the POV^9,11–14^, few studies have used this technique to observe changes in the POV of patients with LSCD or potential limbal damage^11,15^. To the best of our knowledge, no study has used SD-OCT to observe presumed-healthy limbal areas in the eyes with ocular surface disorders or the presumed-healthy fellow eyes of patients with unilateral ocular surface disorders diagnosed by slit-lamp biomicroscopy. In this study, we enrolled patients with advancing wavelike epitheliopathy (AWE) and phlyctenular keratitis/ocular rosacea with total or partial limbal involvement in one or both eyes. We performed slit-lamp biomicroscopy in all patients to define the normal or abnormal patterns of the POV. In addition, we analyzed the POV by using SD-OCT with both tangential and radial cross-sectional scans in the four quadrants of the limbus in both eyes. We determined the power of SD-OCT to evaluate the microstructure of the POV in these patients and found a higher sensitivity of SD-OCT than conventional slit-lamp biomicroscopy in observing the structure of the POV. SD-OCT can provide useful information for monitoring the POV in these patients. The use of SD-OCT can enable early detection of limbal damage in the cornea with potential limbal involvement, leading to proper intervention and prognosis prediction while treating patients with potential LSCD.

## Results

SD-OCT images were obtained from 19 patients. Ten patients were diagnosed as having AWE (a total of 18 eyes, male:female = 2:8, eight patients had bilateral disease and two patients had unilateral disease). Nine patients were diagnosed as having phlyctenular keratitis/ocular rosacea (a total of 15 eyes, male:female = 2:7, six patients had bilateral disease and three patients had unilateral disease). The mean age of patients with AWE and those with phlyctenular keratitis/ocular rosacea was 48.7 ± 14.0 (range: 33–73) years and 30.3 ± 18.2 (range: 9–62) years, respectively. Table 1 summarizes the age, sex, laterality, and quadrants of limbal involvement observed through slit-lamp biomicroscopy of all patients. Of the 18 eyes of patients with AWE (case 1–10), 14 (77.8%) exhibited isolated superior quadrant involvement and 4 (22.2%) had limbal involvement in at least one quadrant, in addition to the involvement of both superior and inferior quadrants. In patients with phlyctenular keratitis/ocular rosacea (cases 11–19), subepithelial infiltration was frequently observed at the leading area of advancing corneal neovascularization in superior or inferior quadrants. Furthermore, of the 15 eyes, 6 (40.0%) showed isolated superior quadrant involvement, 4 (26.7%) showed isolated inferior quadrant involvement, 3 (20.0%) showed combined superior and other quadrant involvement, and 2 (13.3%) showed the involvement of at least one quadrant, in addition to both superior and inferior quadrants.

**Table 1:** Demographic data, disease patterns, and quadrants showing limbal involvement.

Diagnosis	No	Age	Gender	Involved eye	Involved quadrant OD	Involved quadrant OS
Advancing wavelike epitheliopathy (AWE)	1	44	F	OU	Superior	Superior
	2	57	F	OD	Superior	–
	3	36	M	OU	Superior	Superior
	4	34	M	OU	Superior	Superior
	5	33	F	OU	Superior	Superior
	6	41	F	OU	Superior	Superior
	7	73	F	OU	Superior, inferior, nasal, temporal	Superior, inferior, nasal, temporal
	8	62	F	OU	Superior, inferior, nasal, temporal	Superior
	9	44	F	OU	Superior	Superior
	10	63	F	OD	Superior, inferior, nasal, temporal	–
Phlyctenular keratitis/ocular rosacea	11	20	F	OD	Superior, inferior, nasal, temporal	–
	12	22	F	OU	Inferior	Inferior
	13	24	M	OU	Superior, nasal	Superior, temporal
	14	21	F	OD	Superior	–
	15	60	M	OS	–	Superior, inferior, nasal
	16	27	F	OU	Superior, temporal	Superior
	17	28	F	OU	Superior	Superior
	18	9	F	OU	Superior	Superior
	19	62	F	OU	Inferior	Inferior

*M*  male, *F*  female, *No* case number, *OD* right eye, *OS* left eye, *OU* both eyes.

### Abnormal POV was detected through SD-OCT at the lesion sites of the diseased eyes diagnosed by slit-lamp biomicroscopy

**Figure 1:**
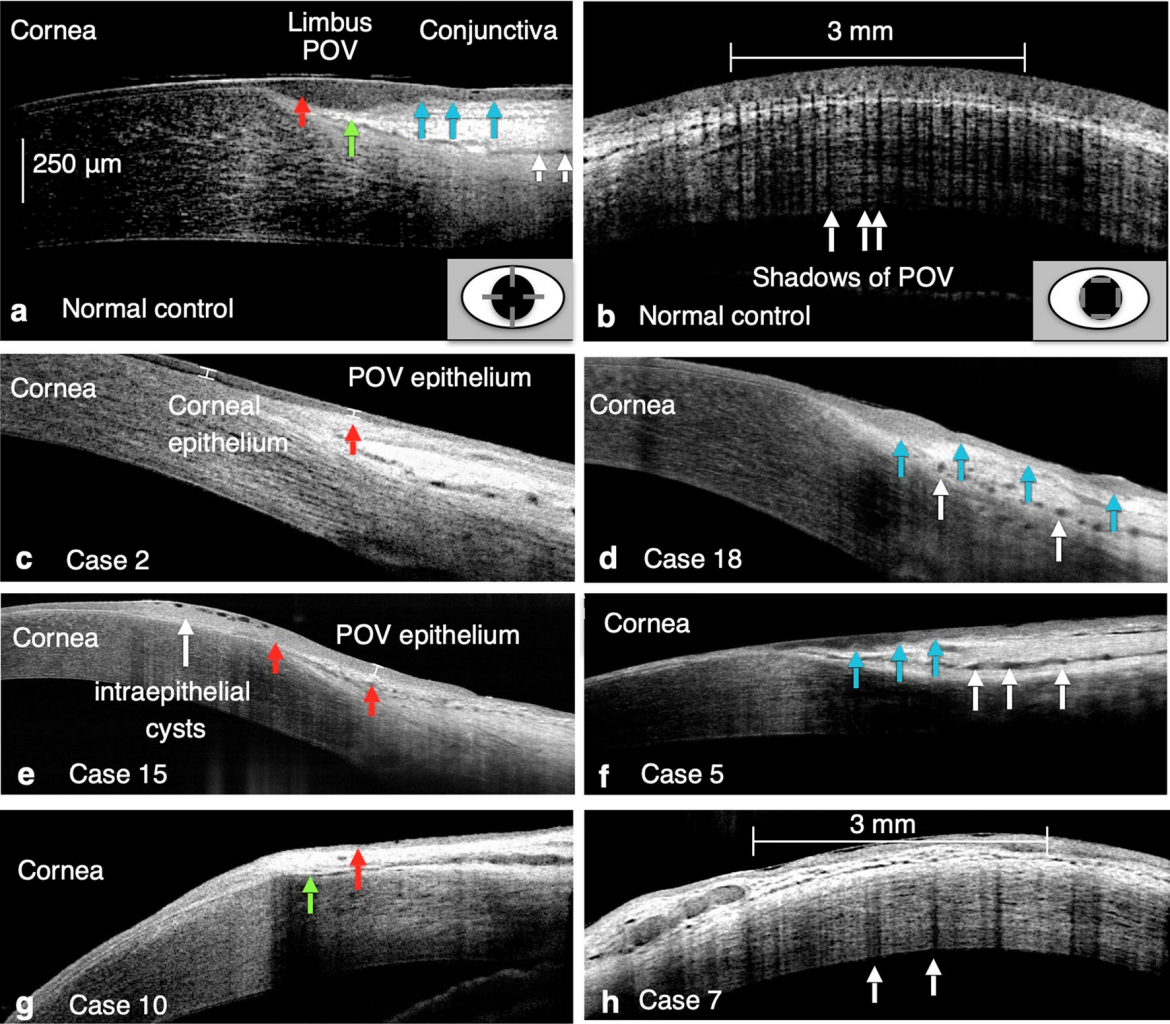
Patterns of the POV defined as “normal” or “abnormal” based on SD-OCT findings. **(a)** The radial cross-sectional scan of a 27-year-old healthy male participant showed following presentations. Red arrow: the maximum epithelial thickness of the POV was at least 1.5-fold greater than the central corneal epithelial thickness, and progressive tapering of epithelial thickness extending to the corneal and conjunctival sides was seen. Green arrow: sharp stromal tip of the POV pointing to the corneal–limbal junction. Blue arrows: a smooth epithelial–stromal interface. White arrows: limbal stromal vessels with lumen diameters < 50 μm. **(b)** The tangential cross-sectional scan showed dense, distinct shadows of the POV with regular intervals in the same participant as **(a)**. The central 3-mm width of scanned images (white line) was calculated. In this photo, the calculated density was 8.3 shadow/mm. White arrows indicate the location and shadows of the POV. The right lower figures in **(a,b)** indicate the scanning area and orientations (gray lines) in radial and tangential cross-sectional scans. **(c)** Representative figure of the decreased epithelial thickness of the POV in the radial cross-sectional scan. The maximum epithelial thickness of the POV (red arrow) was lower than the peripheral corneal epithelial thickness (Case 2, right superior limbus). **(d)** The radial cross-sectional scan showed loss of the smooth epithelial–stromal interface of the POV (blue arrows). Dilated limbal stromal vessels were also found (white arrows) (Case 18, left superior limbus). **(e)** Another figure showing abnormal epithelial thickness of the POV in a radial cross-sectional scan. The epithelium of the POV showed atypical tapering of thickness extending to corneal and conjunctival sides (red arrows). Intraepithelial cysts were also found in this case (Case 15, left nasal limbus). **(f)** Another representative figure of the uneven epithelial–stromal interface of the POV (blue arrows) showed in a radial cross-sectional scan. Dilated limbal stromal vessels were also found (white arrows) (Case 5, left superior limbus). **(g)** Loss of the typical sharp stromal tip of the POV (green arrow) was seen with a decreased epithelial thickness of the POV (red arrow) in a radial cross-sectional scan (Case 10, right superior limbus). **(h)** A tangential cross-sectional scan showed loose shadows of the POV (white arrows) with a calculated density of POV of 2.7 shadow/mm (Case 7, left superior limbus). White arrows indicate the location of POV shadows.

Table 2 summarizes the presentation of the POV in lesion sites according to the five parameters defined in this study (Fig. 1). A decreased epithelial thickness of the POV, loss of the sharp stromal tip of the POV, loss of the smooth epithelial–stromal interface of the POV, dilated limbal vessels, and a decreased density of the POV were observed in 44.4%, 11.1%, 77.8%, 66.7%, and 72.2% of patients with AWE, respectively, and 46.7%, 0%, 40.0%, 86.7%, and 60.0% of patients with phlyctenular keratitis/ocular rosacea, respectively. These SD-OCT findings indicated that limbal involvement in patients with AWE and phlyctenular keratitis/ocular rosacea was not only restricted to the epithelial layer but also involved the limbal stroma and vessels of the POV.

**Table 2:** Presentations of the abnormal POV at the most severe lesion sites of the diseased eyes evaluated through SD-OCT.

Ocular surface disorders	Presentation of abnormal POV (number of eyes)	Percentage (%)
Advancing wavelike epitheliopathy (AWE)	Decreased epithelial thickness (8/18)	44.4
	Loss of sharp stromal tip of POV (2/18)	11.1
	Loss of smooth epithelial–stromal interface (14/18)	77.8
	Dilated stromal vessels (12/18)	66.7
	Decrease density of POV (13/18)	72.2
Phlyctenular keratitis/ocular rosacea	Decreased epithelial thickness (7/15)	46.7
	Loss of sharp stromal tip of POV (0/15)	0.0
	Loss of smooth epithelial–stromal interface (6/15)	40.0
	Dilated stromal vessels (13/15)	86.7
	Decrease density of POV (9/15)	60.0

*POV* palisades of Vogt, *SD-OCT*  spectral-domain optical coherence tomography.

### Abnormal POV was detected through SD-OCT at the presumed-healthy sites of the diseased eyes diagnosed by slit-lamp biomicroscopy

Slit-lamp biomicroscopy findings revealed that 14 of the 18 eyes of patients with AWE and 14 of the 15 eyes of patients with phlyctenular keratitis/ocular rosacea exhibited a presumed-healthy limbus in at least one quadrant. Table 3 lists the percentage of the abnormal POV detected through SD-OCT in the lesion and presumed-healthy sites of the limbus. The abnormal findings was defined according to the five parameters described in the methods as same as those listed in the Table 2. In the AWE group, 100% and 88.9% of the eyes demonstrated at least one and two abnormal findings in the lesion site, respectively, whereas 100% and 64.3% of the eyes exhibited at least one and two abnormal findings in the presumed-healthy sites, respectively. In the phlyctenular keratitis/ocular rosacea group, 100% and 93.3% of the eyes exhibited at least one and two abnormal findings in the lesion site, respectively, whereas 100% and 85.7% of the eyes showed at least one and two abnormal findings in the presumed-healthy site, respectively. Table 4 provides the detailed regional presentations of the abnormal POV detected through slit-lamp biomicroscopy and SD-OCT.

**Table 3:** Percentage of the abnormal POV detected through SD-OCT in the most severe lesion and presumed-healthy sites of the diseased eyes.

Ocular surface diseases	Abnormal POV detected by SD-OCT (number of eyes)	Percentage (%)
Advancing wavelike epitheliopathy (AWE)	**Lesion site (18 eyes)**	
	≥ 1 abnormal finding : 18/18	100.0
	≥ 2 abnormal findings: 16/18	88.9
	≥ 3 abnormal findings: 9/18	50.0
	≥ 4 abnormal findings: 3/18	16.7
	5 abnormal findings: 0/18	0
	**Presumed-healthy site (14 eyes)**	
	≥ 1 abnormal finding : 14/14	100.0
	≥ 2 abnormal findings: 9/14	64.3
	≥ 3 abnormal findings: 7/14	50.0
	≥ 4 abnormal findings: 3/14	21.4
	5 abnormal findings: 0/14	0
Phlyctenular keratitis/ocular rosacea	**Lesion site (15 eyes)**	
	≥ 1 abnormal finding: 15/15	100.0
	≥ 2 abnormal finding: 14/15	93.3
	≥ 3 abnormal finding: 7/15	46.7
	≥ 4 abnormal finding: 3/15	20.0
	5 abnormal findings: 0/15	0
	**Presumed-healthy site (14 eyes)**	
	≥ 1 abnormal finding: 14/14	100.0
	≥ 2 abnormal finding: 12/14	85.7
	≥ 3 abnormal finding: 7/14	50.0
	≥ 4 abnormal finding: 3/14	21.4
	5 abnormal findings: 0/14	0

In total, 4 of the 18 eyes in the AWE group and 1 of the 15 eyes in the phlyctenular keratitis/ocular rosacea group showed limbal involvement in all the four quadrants of the limbal area. Accordingly, there are presumed-healthy sites of the limbus in the 14 disease eyes in both the AWE group and the phlyctenular keratitis/ocular rosacea group.

*POV*  palisades of Vogt, *SD-OCT*  spectral-domain optical coherence tomography.

**Table 4:** Percentage of different abnormal POV patterns in the four quadrants in diseased eyes detected through SD-OCT.

Group	Lesion or presumed-healthy site	Quadrant	No	Decreased epithelial thickness, No (%)	Loss of sharp stromal tips, No (%)	Loss of smooth epithelial–stromal interface, No (%)	Dilated stromal vessels, No (%)	Decrease density of POV, No(%)
AWE	Lesion site	S	18	6 (33%)	2 (11%)	14 (78%)	10 (56%)	13 (72%)
		I	4	1 (25%)	0 (0%)	2 (50%)	3 (75%)	2 (50%)
		N	4	1 (25%)	0 (0%)	4 (100%)	3 (75%)	3 (75%)
		T	4	2 (50%)	0 (0%)	4 (100%)	2 (50%)	3 (75%)
	Presumed-healthy site	S	0	–	–	–	–	–
		I	14	4 (29%)	0 (0%)	7 (50%)	8 (57%)	3 (21%)
		N	14	8 (57%)	0 (0%)	5 (36%)	7 (50%)	3 (21%)
		T	14	5 (36%)	0 (0%)	6 (43%)	6 (43%)	4 (29%)
Ocular rosacea/phlyctenular keratitis	Lesion site	S	11	1 (9%)	0 (0%)	8 (73%)	10 (91%)	6 (55%)
		I	6	5 (83%)	0 (0%)	2 (33%)	4 (67%)	2 (33%)
		N	3	1 (33%)	0 (0%)	3 (100%)	2 (67%)	2 (67%)
		T	3	2 (67%)	0 (0%)	3 (100%)	3 (100%)	3 (100%)
	Presumed-healthy site	S	4	2 (50%)	0 (0%)	1 (25%)	2 (50%)	0 (0%)
		I	9	5 (56%)	2 (22%)	2 (22%)	7 (78%)	2 (22%)
		N	12	3 (25%)	0 (0%)	6 (50%)	10 (83%)	5 (42%)
		T	12	6 (50%)	1 (8%)	9 (75%)	9 (75%)	5 (42%)

*No* case number, *S*  superior limbus, *I*  inferior limbus, *N*  nasal limbus, *T*  temporal limbus.

### Abnormal POV was detected through SD-OCT in the presumed-healthy fellow eyes diagnosed by slit-lamp biomicroscopy

Five patients were found to have unilateral ocular surface disease, which was diagnosed by slit-lamp biomicroscopy (AWE group, cases 2 and 10; phlyctenular keratitis or ocular rosacea group, cases 11, 14, and 15). SD-OCT findings revealed abnormal POV in at least one quadrant in the presumed-healthy fellow eyes of four patients. In patients with unilateral AWE, a decreased epithelial thickness of the POV (case 2), loss of the smooth epithelial–stromal interface of the POV (case 2), dilated limbal vessels (case 2), and a decreased density of the POV (case 2) were detected through SD-OCT. In patients with unilateral phlyctenular keratitis/ocular rosacea, a decreased epithelial thickness of the POV (cases 11, 14, and 15), loss of the smooth epithelial–stromal interface of the POV (case 14), dilated limbal vessels (cases 14 and 15), and a decreased density of the POV (case 14) were detected through SD-OCT. These findings were similar to the abnormal pattern of the POV observed in the presumed-healthy sites of the contralateral diseased eyes.

### Case presentation of AWE

**Figure 2:**
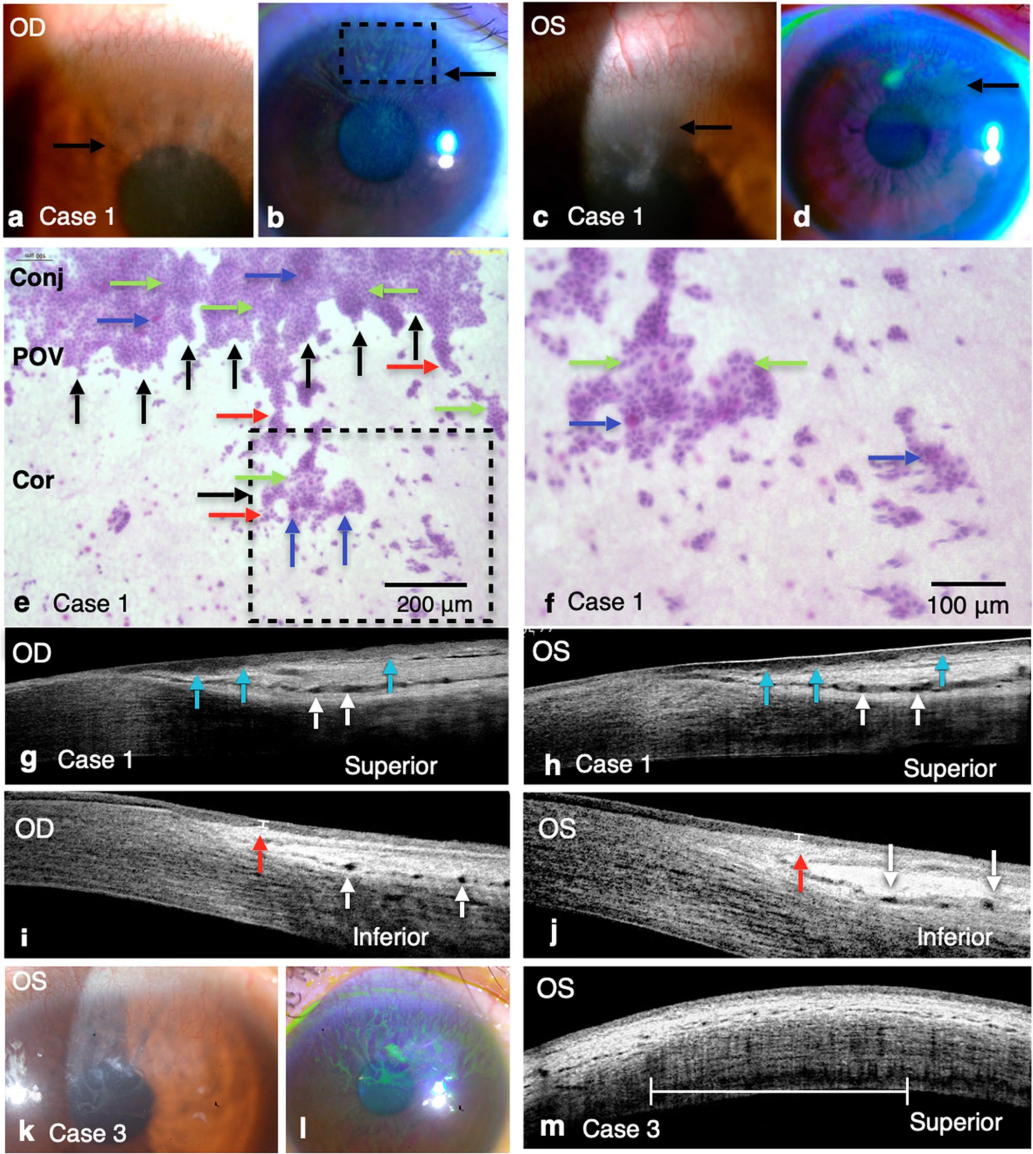
External eye photos, impression cytology, and SD-OCT scans of patients with AWE. **(a–d)** Slit-lamp biomicroscopy demonstrated centripetally advancing waves of the coarse epithelium with fluorescein staining arising from the superior limbus in the right eye **(a,b)** and left eye **(c,d)** of case 1 who was diagnosed as having AWE in the upper quadrant. **(e)** Impression cytology findings of the upper limbus of the right eye in case 1 (square drawn with dotted black lines in **(b)**) showed the central invasion (red arrows) of conjunctival epithelial cells (green arrows) and goblet cells (blue arrows) onto the corneal surface, which is compatible with external eye photos obtained through slit-lamp biomicroscopy. The black arrows indicate the anterior edge of the POV. Compact conjunctival epithelial cells (green arrows) with scattered goblet cells (blue arrows) were found on the conjunctival area (Conj) peripheral to the POV. Limited cells were obtained from the corneal area (Cor). **(f)** The enlarged image of a square drawn with dotted black lines in **(e)**. Green arrows indicate conjunctival epithelial cells, and blue arrows indicate goblet cells on the corneal surface. **(g,h)** Radial cross-sectional scans of SD-OCT at the superior limbus in both eyes of case 1 showed abnormal changes. Loss of the smooth epithelial–stromal interface (blue arrows) with dilated stromal vessels (white arrows) of the POV was found bilaterally. **(i,j)** A radial cross-sectional scan of SD-OCT of the inferior limbus, a presumed-healthy site diagnosed by slit-lamp biomicroscopy, in both the eyes of case 1 showed a decreased epithelial thickness (red arrows) and dilated stromal vessels (white arrows) of the POV. **(k,l)** Slit-lamp biomicroscopy demonstrated centripetally advancing waves of the coarse epithelium with fluorescein staining arising from the superior limbus in the left eye of case 3. Tangential cross-sectional scans of the POV at the superior limbus **(m)** clearly demonstrated the irregular distribution and decreased density of the POV shadow. The calculated density of the POV was 6.7 shadow/mm. The white line indicated the scale bar of 3 mm.

Figure 2a–j shows the external eye photos, impression cytology findings, and SD-OCT scans of a 44-year-old woman with bilateral AWE in the superior limbus (case 1). The external eye photos and impression cytology findings demonstrated the typical advancing waves of conjunctival epithelial and goblet cells migrating onto the corneal surface at the superior quadrant. The radial cross-sectional OCT scan of the superior limbus in both the eyes showed loss of the smooth epithelial–stromal interface and dilated stromal vessels of the POV. Although no evidence of limbal involvement diagnosed by slit-lamp biomicroscopy was observed in other quadrants, a decreased epithelial thickness and dilated stromal vessels of the POV were observed in the inferior limbus (presumed-healthy sites) in both the eyes through SD-OCT. Figure 2k–m demonstrates another representative case of AWE who was a 36-year-old man (case 3) with pathology at the superior limbus in both the eyes. The tangential cross-sectional scan demonstrated a decreased density of the POV in the superior limbus.

### Case presentation of ocular rosacea

**Figure 3:**
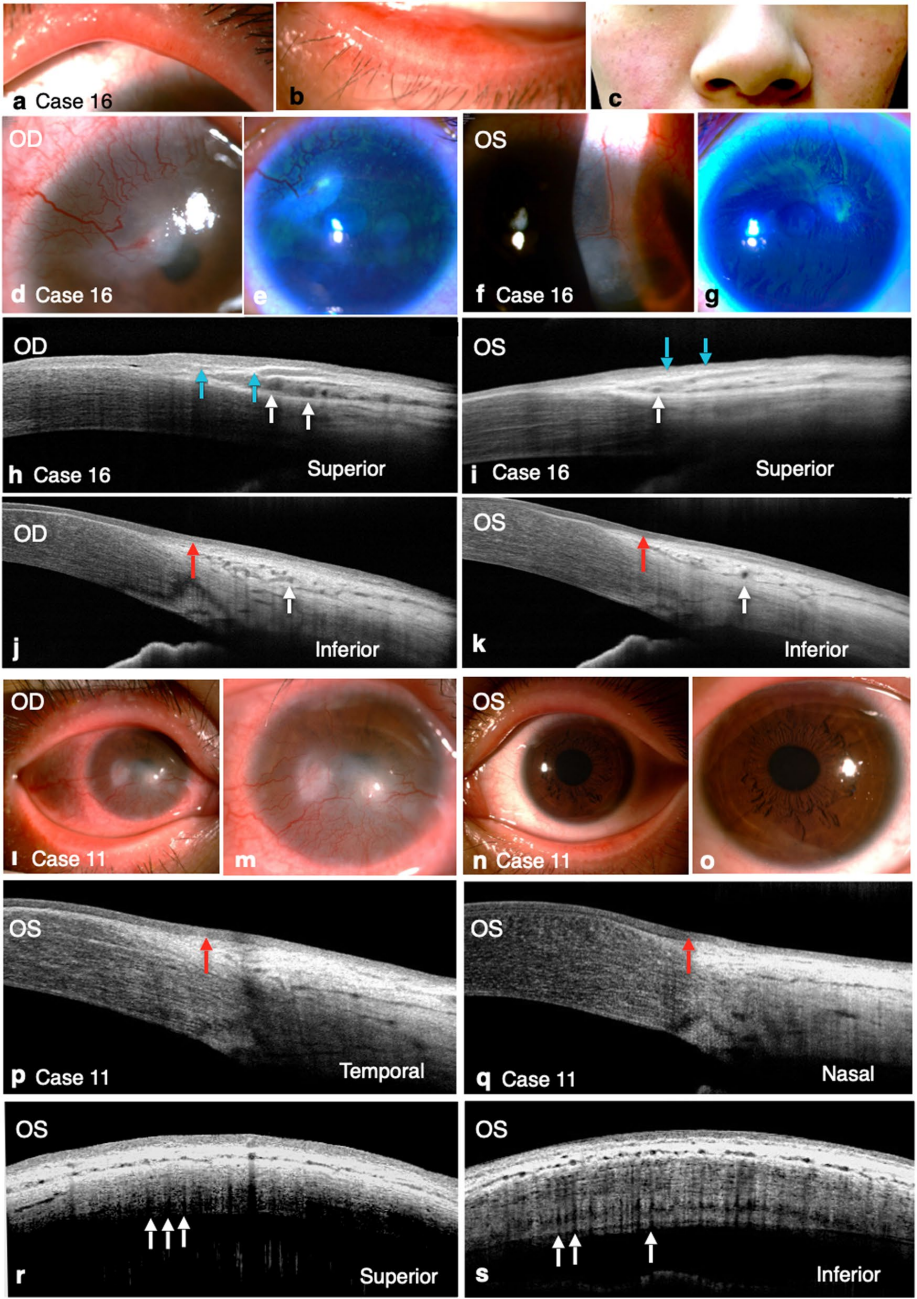
External eye photos and OCT scans of patients with ocular rosacea. **(a–c)** Obstruction of meibomian glands in the upper and lower eyelids and facial erythema were found in case 16, a 27-year-old female patient with bilateral ocular rosacea. **(d–g)** In this case (case 16), subepithelial infiltration along the advancing vascular border from the superior temporal and superior limbus was found in the right eye **(d,e)** and left eye **(f,g)**. **(h–k)** The radial cross-sectional scans of SD-OCT of the superior and inferior limbus in both the eyes. Blue arrows: An unsmooth epithelial–stromal interface. White arrows: Engorged stromal vessels with enlarged lumen diameters. Red arrow: The decreased epithelial thickness of the POV. **(l–o)** Case 11 of ocular rosacea. Prominent obstruction of the meibomian gland and corneal neovascularization with subepithelial infiltration mainly in the lower half of the cornea were found in the right eye. However, neovascularization from limbus was found in all quadrants **(l,m)**. The left eye was diagnosed to be “presumed-healthy” through slit-lamp biomicroscopy **(n,o)**. **(p–s)** SD-OCT of the presumed-healthy left eye **(p,q)** showed a decreased epithelial thickness of the POV and **(r)** a decreased density of the POV in the upper limbus. Red arrow: a decreased epithelial thickness of the POV. White arrows: the location of POV shadows.

Figure 3a–k shows the eye photos of a 27-year-old woman with bilateral ocular rosacea (case 16). Typical findings of ocular rosacea, including facial erythema and bilateral meibomian gland obstruction, were found. Corneal neovascularization and infiltration in the superior and temporal limbus extending to the cornea were detected through slit-lamp biomicroscopy. The involved area also exhibited an unsmooth corneal surface, as observed through fluorescein staining. However, slit-lamp biomicroscopy showed no pathological change in the inferior limbus. Furthermore, SD-OCT findings revealed loss of the smooth interface between the epithelium and stroma and dilated stromal vessels of the POV in the superior limbus in both the eyes. A decreased epithelial thickness and dilated stromal vessels of the POV were also observed in the inferior limbus, and these were diagnosed as presumed-healthy sites through slit-lamp biomicroscopy. Figure 3l–s demonstrates another case of unilateral ocular rosacea (case 11, a 20-year-old woman). In this patient, slit-lamp biomicroscopy showed prominent meibomian gland obstruction and diffuse corneal neovascularization with subepithelial infiltration in the right eye mainly in the lower half of the cornea. No corneal pathology was noted in the left eye (presumed-healthy eye) through slit-lamp biomicroscopy. However, some abnormal patterns of the POV were found in the left eye, including decreased epithelial thickness and decreased density of POV.

## Discussion

The POV are a series of radially oriented fibrovascular ridges, similar to fingerprints, located in a 1–2 mm band of the corneoscleral limbus. In this area, the epithelium of the POV projects downward between the subepithelial papillae. Usually, the POV structure could be identified through slit-lamp biomicroscopy in only 20% of the healthy eyes^16,17^^.^ In patients in whom the POV could not be detected through slit-lamp biomicroscopy, the limbus was still diagnosed as “healthy” if no perilimbal injection, swelling, infiltration, ischemia, degeneration, malignancy, or neovascularization was found. IVCM is a powerful technique that has been used to visualize cells located in the POV in the past decades^17–20^. However, in IVCM, the requirement of direct contact with the eye is a disadvantage for patients with ocular surface disorders. AS-OCT is a rapid, noninvasive, highly repeatable modality that can provide high-resolution images. In the past years, optical coherence tomography (OCT) has become a widely used clinical tool for imaging of the ocular surface, although it was mostly used for the observation of the optic disc and retinal choroidal structure^21^. Recently, studies have used AS-OCT to visualize the POV in healthy^9,13^ and pathological conditions^11–13^. Although SD-OCT has not yet been able to identify the structure of individual limbal stem cells, research-grade OCT with a high resolution (0.95-μm axial resolution and 2-μm lateral resolution) could visualize individual pigmented cells along fibrous folds and red blood cells inside the capillaries of the POV^22^. In addition, Chen et al. reported that full-field OCT could provide both cross-sectional and en face images of the POV at a submicron resolution, and even the corneal nerve could be clearly observed^23^. Lathrop et al. used SD-OCT to identify the POV and reported an excellent structural correlation with images obtained through IVCM^24^. Banayan et al. reported the 3D structure of the POV with various scan orientations of SD-OCT (parallel, perpendicular, and en face), and both the stromal and epithelial components could be identified^12^. All these evidence demonstrate the potential of SD-OCT in evaluating patients with ocular surface disorders.

In this study, we proposed five simple but reliable qualitative and quantitative parameters for the radial and cross-sectional images of the POV obtained through SD-OCT: (1) a decreased epithelial thickness, (2) loss of the sharp stromal tip, (3) loss of the smooth interface between the epithelium and stroma, (4) dilated stromal vessels, and (5) a decreased POV density. Using these five parameters, we evaluated the possibility of detecting subclinical changes in the POV through SD-OCT before detection through slit-lamp biomicroscopy. We focused on two types of ocular surface disorders, namely AWE and phlyctenular keratitis/ocular rosacea, which typically involve the limbal areas. We evaluated four quadrants of the limbal area through tangential and radial cross-sectional scanning. According to the result of our previous study^9^, the positive rate of typical POV was higher in superior limbus (68–100%) and inferior limbus (25–100%) while relatively lower in nasal limbus (0–69%) and temporal limbus (4–65%). The majority of the study subjects presented with lesions located in the superior or inferior limbus. As the nasal or temporal limbus was evaluated as presumed-healthy sites, the presence of atypical patterns of POV was predicted to be higher. In the current study, the slightly higher percentage of > / = 3 abnormal features might not be considered as a presentation of an earlier phase of the disease.

In this study, we found a high detection rate of abnormal POV by using SD-OCT on those limbal areas in which slit-lamp biomicroscopy clearly demonstrated pathological changes in both disease groups. SD-OCT provided information regarding epithelial thickness, epithelial–stromal interface, subepithelial stromal morphology, and stromal vessel diameters in the POV. SD-OCT could also detect the density of POV with high reliability and repeatability^10,22^; compared with slit-lamp biomicroscopy, SD-OCT was more sensitive and useful in examining the health of limbal microstructure. To the best of our knowledge, information provided by our study has not been reported thus far. Furthermore, a higher detection rate of abnormal changes in the POV by SD-OCT was demonstrated through recognition of subclinical pathologies in presumed-healthy sites of the diseased eyes or presumed-healthy fellow eyes previously diagnosed by slit-lamp biomicroscopy.

These subclinical changes in the POV included changes in the epithelial thickness, an unsmooth interface between the epithelium and stroma, loss of the typical stromal figure, and dilated stromal vessels. Our results suggested that SD-OCT can serve as a useful noninvasive tool for evaluating clinical and subclinical changes in the limbal area in ocular surface disorders and provide higher-quality images compared with those obtained using current technologies. Such high detectability is particularly useful for monitoring partial or subclinical LSCD, eventually aiding in the administration of appropriate treatment. In addition to AWE and phlyctenular keratitis/ocular rosacea, patients with other potential limbal damage can be benefited by SD-OCT, including those wearing contact lenses and those with chemical burns, topical drug toxicity, and Stevens–Johnson syndrome^25–30^.

This study has some limitations. First, SD-OCT image resolution was compromised by the instability of eye fixation toward nonprimary gaze, particularly in patients with severe ocular surface disorders who were sensitive to light. Therefore, careful and adequate application of topical anesthetic eye drops before an examination is required. Second, the site of the radial cross-sectional scans of the POV differed from the interpalisade to palisade area, whereas that of the tangential cross-section scan deviated between the corneal and scleral side. Repeated examination by experienced technicians or doctors is required. Third, the case number is not large enough and not all patterns of ocular surface disorders were included in this study. Fourth, we combined patients with phlyctenular keratitis and ocular rosacea in the same group due to difficulty in the differential diagnoses in some patients, particularly in young adults or children. However, despite the aforementioned limitations, we found some novel results of the structural change of the POV in patients with AWE and phlyctenular keratitis/ocular rosacea by using SD-OCT. With new sub-micrometer axial resolution optical coherence tomography, the high spatial OCT resolution allowed for precise identification of POV by visualization of the capillaries that extend from the inside of the palisade’ ridges to a lateral micro-vascular network located underneath the POV^22,23^. Extension of the study method to other ocular surface disorders can be a safe and valuable future clinical application.

In conclusion, the POV and limbus structure could be effectively visualized through SD-OCT, and this imaging modality can be used to detect subclinical changes in the POV in the presumed-healthy sites of the eyes in patients with ocular surface disorders. These findings helped us gain novel insights into the management of ocular surface disorders, and can aid in diagnosis, monitoring, treatment-related decision making, and prognosis prediction in clinical practice.

## Methods

In this cross-sectional, observational study, we included 18 eyes of 10 patients with AWE (2 male and 7 female; age range: 33–73 years) and 15 eyes of 9 patients with phlyctenular keratitis or ocular rosacea (2 male and 7 female; age range: 9–62 years). Slit-lamp biomicroscopy was used for the diagnosis. In AWE, slit-lamp biomicroscopy revealed centripetally advancing waves of the wavy, coarse, irregular epithelium extending from the limbus to the central cornea. In phlyctenular keratitis, corneal phlyctenules presented as round, elevated, gray, or yellow nodules accompanied by a zone of engorged hyperemic vessels emerging from the limbal area. Some degree of ulceration and fluorescein staining was found with the progression of corneal phlyctenules. In ocular rosacea, bilateral telangiectasia and inspissation or obstruction of the meibomian glands associated with typical rosacea facial lesions were observed under the slit-lamp biomicroscope. We enrolled only patients who presented with manifestation of corneal inflammation, including peripheral stromal infiltration, opacification, scarring, or neovascularization, in addition to the typical findings of meibomian gland diseases and facial lesions. We combined patients with phlyctenular keratitis and ocular rosacea in the same group (phlyctenular keratitis/ocular rosacea) due to difficulty in the differential diagnosis in some patients, particularly young adults or children.

The study was performed in the Department of Ophthalmology, National Taiwan University Hospital, from Jun, 2018 to Jun, 2019. This study was conducted in accordance with the Declaration of Helsinki and approved by the Institutional Review Board of National Taiwan University Hospital (ClinicalTrials.gov ID: NCT03594370). After explaining the study purpose, we obtained written informed consent from all patients. For one participant under the age of 18 years, informed consent had been obtained from the parent and legal guardian. Figure 3c includes the facial image that may lead to identification of the study participant, informed consent had been obtained to publish the image in an online open-access publication. Patient names were removed from all text/figures/tables/images.

All patients underwent slit-lamp examination and SD-OCT examination at the outpatient department. The SD-OCT examination was performed as reported in a previous study^9^ with some modifications as follows. We evaluated all four quadrants of the limbal area through tangential and radial cross-sectional scanning and ensured that the POV area could be clearly seen. Superior, inferior, nasal, and temporal areas were routinely examined. However, oblique orientations were examined in some cases if the most severe part of the limbal pathology was not located in the superior, inferior, nasal, or temporal area. Regardless of the extent of AWE, phlyctenular keratitis, or ocular rosacea, quadrants exhibiting the most severe pathological change were considered the lesion sites. Areas presumed to be healthy on the eyes with lesions or the fellow eyes presumed to be healthy through slit-lamp biomicroscopy were carefully reviewed, and quadrants exhibiting the most severe pathological change, as revealed by SD-OCT findings, were considered the presumed-healthy sites or presumed-healthy eyes, respectively.

A SD-OCT system (RTVue-100; Optovue Inc., Fremont, CA, USA) with a noncontact cornea anterior module was used for the analysis. This system was operated at a wavelength of 840 nm. The scan speed was 26,000 axial scans per second. The width and depth of the scan ranged from 2 to 12 mm and 2 to 3 mm, respectively. The depth of the resolution (axial resolution) was 5 μm in the tissue, whereas the transverse resolution was 15 μm^9^. Adaptor lenses (wide-angle long corneal adaptor lens) were used in this study and provided the scan with a length of 6-mm- and a depth of 1.96-mm^14^. Radial and tangential cross-sectional scans were obtained simultaneously. To obtain the accurate pattern and the maximum epithelial thickness of the POV, patients were asked to keep looking at the four directions to maintain the perpendicularity of the OCT beam at the surface of the targeted tissue. To compare the epithelial thickness between the POV and central cornea, we obtained the cross-sectional scans of the central cornea.

Regarding the POV structure, we analyzed epithelial thickness, stromal configuration, interface contour between the epithelium and stroma, limbal vessel diameter, and POV density (numbers/mm) in the tangential cross-section scan (Fig. 1). In this study, the normal pattern of the POV evaluated through SD-OCT was defined using our previous method with some modifications^9^. In radial cross-sectional scans, the following four parameters were considered the normal pattern of the POV: (1) the maximum epithelial thickness of the POV should be at least 1.5-fold greater than the corneal epithelial thickness, (2) the stroma of the POV should be presented as a sharp tapering tip pointing to the corneal–limbal junction, (3) a smooth and clearly identified interface should be observed between the epithelium and stroma, and (4) the lumen diameter of the limbal vessels should be < 50 μm. We added another parameter; that is, the density of the POV, for the tangential cross-sectional view. The POV was considered to have normal density if we observed dense, distinct shadows of the POV with an average density of > 8.0 shadow/mm^22^.

The followings were the five morphological criteria used to determine whether the POV was abnormal or not in this study, including (1) POV epithelial thickness thinner than the corneal epithelial thickness, (2) loss of the sharp stromal tip, (3) loss of the smooth interface between the epithelium and stroma, (4) dilated stromal vessels with diameter > 50 μm, and (5) POV density < 7.0 shadow/mm. The cut off value of the first parameter was based on the study^9^ conducted previously by our team, which revealed the central corneal epithelial thickness was 51.33 μm and 51.67 μm in the group aged 20–39 and 40–59 respectively. The maximum epithelial thickness of POV was 106.9 ± 17.0 μm, 106.2 ± 22.8 μm, 85.6 ± 13.2 μm and 82.8 ± 13.1 μm at superior, inferior, temporal and nasal limbus respectively. Accordingly, the normal pattern of POV has been established with the maximum epithelial thickness of POV at least 1.5 thicker than the central corneal epithelial thickness in the previous study. And the difference of peripheral corneal epithelial thickness and central epithelial thickness is minimal. Thus, we referred to the peripheral corneal epithelial thickness for direct comparison in each OCT scan to evaluate the first and the fourth criteria. At each limbal area, those radial cross-sectional images with high quality which can clearly show the interface of epithelium-stroma were selected for further analysis. For those tangential cross-sectional scans, the best quality scan was obtained by a trained operator based on the visibility and contrast of the line pattern corresponding with the POV. The tangential cross-sectional scans consisted of the best presented shadows of POV (usually > 10 shadows^10^ in one scan in the presumed-healthy areas of the diseased eyes, and the presumed-healthy eyes diagnosed by slit-lamp biomicroscopy) in the scan were selected for further analysis of palisade density. In case without clearly identified shadow of POV, slit-lamp findings were also used to verify the absence of POV. The density of the POV was calculated three times within the central 3-mm width of scanned images in the tangential cross-sectional view where the shadows of the POV were clearly identified^14^. The vertical shadows underneath the highly reflective, narrow, loop-like structures of POV and projecting through the whole length of cornea were counted.

All the images were evaluated by two examiners (Y-YC and Y-CS) individually and finally approved by the third examiner (W-LC) to avoid the inter-observer variability. When there was discrepancy between the first two evaluations, the assessment of a third examiner was decisive. All the examiner was unaware of the study subject of the OCT scan under interpretation.

In patients with AWE, we performed impression cytology on the lesion site to confirm the presence of conjunctival invasion, which is a typical presentation of LSCD. Samples were collected from four limbal quadrants using cellulose acetate filter papers (Millicell CM; 0.4 μm; Millipore Corporate, Billerica, MA, USA) in both the eyes. After applying one drop of the topical anesthetic (0.5% proparacaine hydrochloride, Alcaine, Alcon-Couvreur, Puurs, Belgium), excess tears were gently removed using cotton swabs. The cellulose acetate filter paper was placed on the surface of the bulbar conjunctiva, limbal area, and peripheral cornea. After applying slight pressure for 30 seconds, the sampling filter paper was removed from the surface and immediately stored in 95% alcohol. The samples were stained with periodic acid-Schiff and hematoxylin stains and photographed at 100 × and 200 × magnifications. All limbal impression cytology specimens were obtained and evaluated by the same technician.

## Abbreviations

AWE: Advancing wavelike epitheliopathy
SD-OCT: Spectral-domain optical coherence tomography
AS-OCT: Anterior segment optical coherence tomography
IVCM: In vivo confocal microscopy
LSCD: Limbal stem cell deficiency
OCT: Optical coherence tomography
POV: Palisades of Vogt
